# Behaviour, use and safety aspects of astatine-211 solvated in chloroform after dry distillation recovery

**DOI:** 10.1038/s41598-024-60615-4

**Published:** 2024-04-27

**Authors:** Emma Aneheim, Ellinor Hansson, Chiara Timperanza, Holger Jensen, Sture Lindegren

**Affiliations:** 1 Department of Medical Radiation Sciences, Institute of Clinical Sciences, Sahlgrenska Academy, University of Gothenburg, 41345 Gothenburg, Sweden; 2 Department of Oncology, Region Västra Götaland, Sahlgrenska University Hospital, 41345 Gothenburg, Sweden; 3 Atley Solutions AB, 41327 Gothenburg, Sweden; 4 Department of Clinical Physiology and Nuclear Medicine, Cyclotron and Radiochemistry unit, Copenhagen University Hospital, 2100 Copenhagen, Denmark

**Keywords:** Chemistry, Nuclear chemistry

## Abstract

Targeted alpha therapy of disseminated cancer is an emerging technique where astatine-211 is one of the most promising candidate nuclides. Astatine-211 can be produced in medium energy cyclotrons by alpha particle bombardment of natural bismuth. The produced astatine is then commonly recovered from the irradiated solid target material through dry distillation. The dry distillation process often includes elution and solvation of condensed astatine with chloroform, forming Chloroform Eluate. In this work the handling and safe use of the high activity concentration Chloroform Eluate has been investigated. Correctly performed, evaporation of Chloroform Eluate results in a dry residue with complete recovery of the astatine. The dry residue can then serve as a versatile starting material, using appropriate oxidizing or reducing conditions, for subsequent downstream chemistry. However, it has been found that when evaporating the Chloroform Eluate, astatine can be volatilized if continuing the process beyond the point of dryness. This behavior is more pronounced when the Chloroform Eluate has received a higher absorbed dose. Upon water phase contact of the Chloroform Eluate, a major part of the astatine activity becomes water soluble, leaving the organic phase. A behavior which is also dependent on dose to the solvent.

## Introduction

Astatine-211 is an alpha particle emitting radionuclide relevant for nuclear medicine applications through so called targeted alpha therapy (TAT). Alpha particles have a short range in tissue (< 100 μm) and high energy, resulting in both a high linear energy transfer and relative biological effectiveness^1,2^. This makes TAT a suitable method for treatment of disseminated cancer in the form of micro-tumors or single cancer cells. In TAT the radioactive nuclide is attached to a biological targeting vector, targeting specific or over-expressed structures or functions of the tumor cells. The carrier vector can be, *e.g*. a small molecule, a peptide or a protein, such as a monoclonal antibody^3^.

Astatine-211 has a half-life of 7.2 h and is most commonly cyclotron produced from natural bismuth through an alpha, 2n reaction, ^209^Bi(α, 2n)^211^At. Significant amounts, up to 9 GBq, can be produced using a solid target system^4^. Dry distillation is the predominant method used for astatine recovery from such irradiated solid targets. This process is utilizing the low boiling point of astatine, T_b_ < 400 °C^5^, compared to the bismuth starting material, T_b_ = 1564 °C, and any commonly used target backing materials such as aluminium, T_b_ = 2519 °C or copper, T_b_ = 2560 °C, hence separating gaseous astatine from the other materials upon heating^6^. Astatine-211 is a halogen, with certain metallic characteristics, and is therefore readily soluble in organic diluents. Common practice at several different locations world-wide, such as Nantes-France, Gothenburg-Sweden and Fukushima-Japan, that all apply dry-distillation, is to solvate the separated astatine in chloroform.

From the teams in Nantes particularly, a lot of important fundamental data on Astatine has been reported lately^7^. However, alpha particle emission, especially under clinically relevant activity concentrations, induce severe radiolysis of diluents, such as chloroform, where the nuclide is solvated^8^. Due to this, much of the fundamental data on astatine does not apply under high activity concentration conditions in solution. In the case of astatine, being a halogen, this also mean that astatinated degradation products in the form of small organic molecules or di-halogens, with unknown properties, can be formed.

Historically, few research labs around the world have worked with astatine because of its short half-life that requires relatively close proximity to a medium energy cyclotron, capable of accelerating alpha particles of *circa* 30 MeV, for producing astatine^9^. In Gothenburg the Targeted Alpha Therapy research group has been working with astatine since 1994 including basic chemistry, preclinical work and a clinical study that was conducted 2005–2012, applying the astatinated Fab2-fragment MX-35 for treatment of ovarian carcinoma^10,11^. However, there is no cyclotron in Gothenburg capable of producing astatine and therefore the nuclide had to be produced elsewhere. Early on this was done in Oslo and Rossendorf but since 2002 the supply of astatine-211 has been provided by the Scanditronix MC32 cyclotron at the Copenhagen University Hospital in Denmark. Since the start in Gothenburg, dry distillation has been the method of choice for astatine recovery from the irradiated target and chloroform used as the preferred diluent for solvating astatine^12^.

During the last years, a significant global increase in interest around using astatine-211 for TAT has emerged, which has led to a number of new research projects being started. Currently several clinical Phase I trials in USA and Japan, are ongoing with this promising nuclide, targeting different types of cancers^13^. This has led to an increased interest from both academic and industrial research groups without prior experience in working with astatine. They are now starting to look for clinical applications, which means working with significant amounts of activity. To meet the requirements for clinical trials with astatine-211, using proper methods from irradiated target to the finished radiopharmaceutical is essential.

Previously we have initiated investigations of the high activity concentration chloroform solution with solvated astatine, resulting from work-up after dry distillation of the irradiated target^14^. In the present study we have continued this work, focusing on handling and use of the astatine-211 chloroform solution in a safe and efficient way. This includes safe and efficient evaporation of chloroform containing astatine-211 as well as differentiating astatine forms upon aqueous contact and during interactions with oxidizing- and reducing agents for downstream chemistry.

## Results

### Evaporation of chloroform eluate

**Figure 1:**
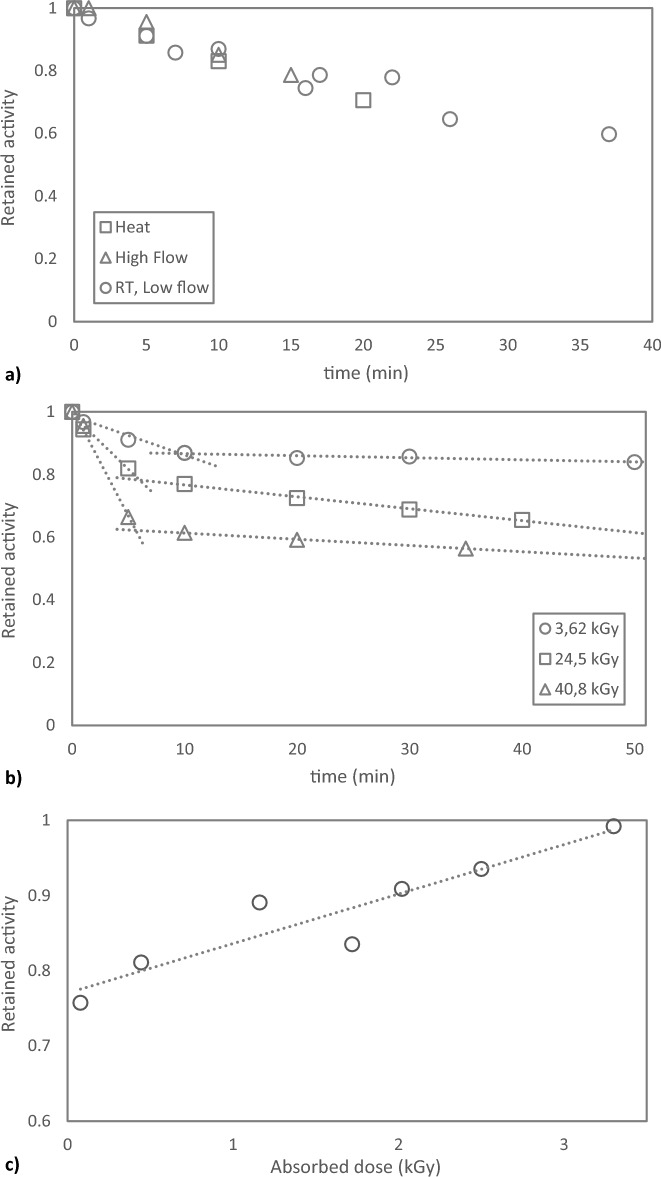
Evaporation of Chloroform Eluate. (**a**) Remaining activity upon continued flow of N_2_ (g) on a dry ^211^At residue after evaporation of Chloroform Eluate. Each point represents one experimental value derived at n = 5 experimental occasions with absorbed dose 2.5–13 kGy. Flow for datapoints room temp and heat 130 mL min^−1^, high flow 210 mL min^−1^. Room temp = 20 °C, High temp = 46 °C. (**b**) Remaining activity upon long-time continued gentle flow of N_2_ (g) after evaporation of Chloroform Eluate to dryness with one Chloroform Eluate batch that has received different absorbed doses before the evaporation process. (**c**) Direct evaporation of Chloroform Eluate, formed using chloroform stored in a clear glass container for > 3 weeks, to the point of dryness using a gentle N_2_ flow. Each point represents one experimental value derived at n = 5 experimental occasions.

The resulting astatine solution in chloroform, following dry distillation recovery, will in this work from now on be termed Chloroform Eluate. It has previously been shown that Chloroform Eluate can be evaporated to dryness in an open vial with a gentle flow of nitrogen gas, N_2_, resulting in a complete activity retention^14^. However, in the current study, this has only been found true if the nitrogen flow is removed directly upon visual dryness. A continuous gentle N_2_ flow of 130 mL min^−1^ at room temperature over a dry vial with astatine following chloroform evaporation induces a significant loss of activity as is evident in Fig. 1a (data from n = 3 experimental occasions). Similarly, applying heat (40–60 °C) or increasing the flow of N_2_ (to 210 mL min^−1^) upon evaporation of Chloroform Eluate show a complete retention of astatine activity upon termination directly after reaching dryness. In the case of an extended N_2_ flow beyond the point of complete evaporation, a similar loss of activity as with the gentle N_2_ flow at room temperature is induced in both cases, Fig. 1a. However, if an extreme N_2_-flow is applied (up to 800 mL min^−1^) then activity losses of ca 5% can be observed even if terminating the evaporation only a few seconds after reaching dryness, as could be expected. In a separate experiment, one single batch of Chloroform Eluate was subjected to a prolonged nitrogen flow beyond the point of solvent dryness and the result put into relation to the specific absorbed dose to the chloroform prior to the evaporation process, Fig. 1b. An increased absorbed dose by the Chloroform Eluate resulted in a primary steeper slope of the linear loss of activity from the dry residue. In addition, the prolonged nitrogen flow reveals that the activity loss reaches a plateau, which is reached quicker, but at a lower level, for higher absorbed doses. This results in a more significant loss of activity, as compared to chloroform that has received a lower absorbed dose. These findings confirm the previously postulated formation of several different chemical forms of astatine in chloroform upon irradiation^14^ but also indicate that these forms have different vapor pressure. Evaporation after > 24 h of Chloroform Eluate, having received a cumulated very high dose (32–41 kGy), still show no loss of activity with 100% astatine retention, stopping the gentle evaporation process upon dryness.

Leaving a vial with a dry residue from evaporated Chloroform Eluate without manipulation ensures that the astatine activity remains, if the vial is provided with a lid. Opening the lid for 60 s intervals on several occasions throughout one half-life does not affect this result. However, from dry vials left without a lid in an environment with significant turn-over of the atmosphere, such as a fume hood, activity is slowly leaving (*circa* 1%/h), as could be expected from the results presented in Fig. 1.

Using aged chloroform (stored in clear glass and exposed to light for > 3 weeks) in the work-up after dry distillation, i.e. to form Chloroform Eluate, was observed to severely impact activity stability upon subsequent solvent evaporation to dryness. Losses up to 24% was observed without any excess gas flow beyond the point of dryness. A clear relation between absorbed dose to solvent and loss of activity could also be seen, where an absorbed dose over *circa* 3.3 kGy resulted in negligent losses, Fig. 1c. The absorbed dose of this Chloroform Eluate is a consequense of the astatine activity entering the dry distillation process and subsequent process yield, the solvent volume applied in said dry distillation process and the time after recovery before starting the evaporation. Using fresh chloroform from the original container to form Chloroform Eluate showed no loss of activity upon evaporation to dryness even at as low doses as 0.7 kGy (evaporation efficiency > 99% for 3 evaporations). At this dose level, Chloroform Eluate formed using aged chloroform, stored in clear glass, showed an activity loss of > 15% upon evaporation to dryness.

In previous work it was postulated that the presence of halogens in form of chlorine in the solvent was contributing to the stability of Chloroform Eluate upon evaporation^14^. In order to investigate if it would be possible to replace chlorine based solvents with brominated solvents, di-bromo methane was added to a dry residue of astatine and allowed to react before being subjected to drying. In this case a loss as of activity as high as 40% was observed after a contact time of 20 min before evaporation to dryness. Contrary to what was previously seen for chloroform and di-chloro methane^14^, an increased contact time and hence absorbed dose to the solvent also negatively affected the stability, Supplementary Fig. S1.

### Chloroform eluate and aqueous phase contact

**Figure 2:**
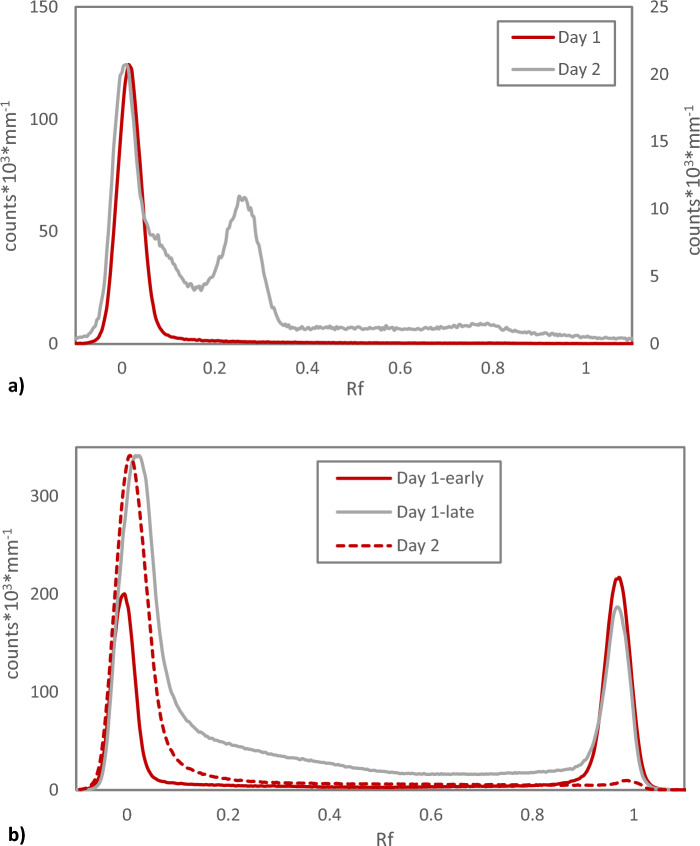
Radio-TLC of Chloroform Eluate. (**a**) Reversed Phase TLC of Chloroform Eluate Day 1 at 4 h after elution (absorbed dose 11.1 kGy) compared to the chloroform eluate Day 2 at 24 h after elution (absorbed dose 31.4 kGy). (**b**) Normal Phase TLC of Chloroform Eluate Day 1 at 0.5 and 8 h after elution (absorbed dose 1.2 and 13.8 kGy respectively) compared to the Chloroform Eluate Day 2 at 26.5 h after elution (absorbed dose 33.7 kGy).

**Figure 3:**
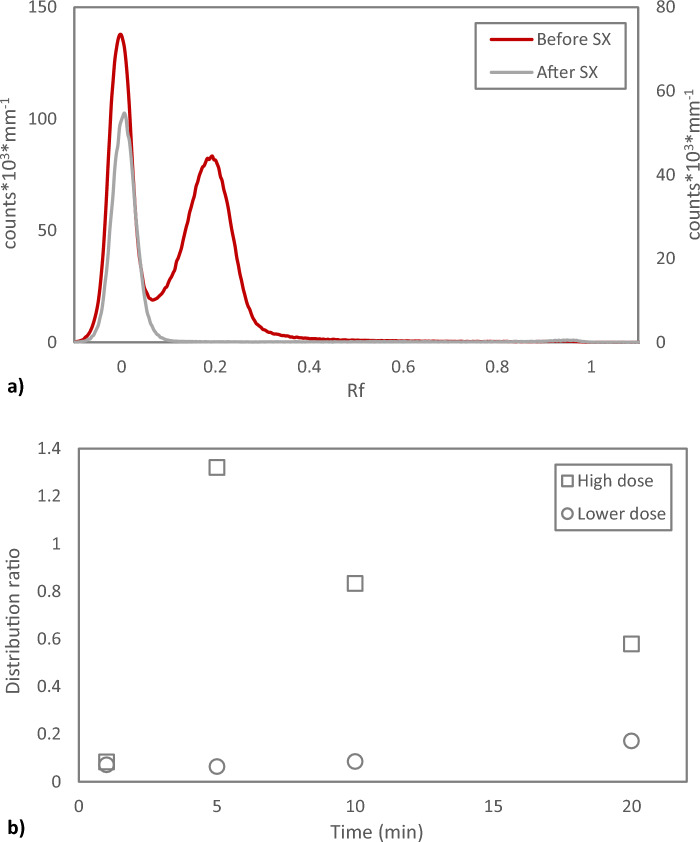
Solvent extraction of Chloroform Eluate. (**a**) Reversed phase radio-TLC of Chloroform Eluate with high absorbed dose (38.8 kGy) before and after solvent extraction (SX) with equal phase volumes of 0.1 M NaClO_4_ for 20 min. (**b**) Kinetics of solvent extraction of Chloroform Eluate with high absorbed dose (38.8 kGy) as well as lower absorbed dose (8.9 kGy). Equal phase volumes and aqueous phase 0.1 M NaClO_4_.

As already mentioned above, Chloroform Eluate can be evaporated without activity loss even when receiving a very high absorbed dose (up to 41 kGy) after long-time radiation exposure. However, when analysing such Chloroform Eluate with TLC, it is evident that the chemical form(s) of astatine changes as compared to more fresh Chloroform Eluate. On Reversed Phase TLC, a second peak with a possibly more polar form of astatine appears besides the one(s) at Rf = 0. In line with this result, on Normal Phase TLC, the two peaks with different mobility in the system, caused by e.g. difference in polarity and/or different reactivity towards the Silica, present at low doses (Rf = 0 and Rf = 1) gradually changes into one single peak at Rf = 0, Fig. 2. In previously reported Reversed Phase HPLC data, for lower absorbed doses, a relation between absorbed dose of Chloroform Eluate and activity retention on the HPLC column could be found, with an almost complete retention of activity on the column for higher absorbed doses^14^. This in combination with the results presented herein shows that TLC analysis could be a more suitable method for direct comparative analysis of Chloroform Eluate. Subjecting an old Chloroform Eluate to solvent extraction with a non-complexing aqueous phase (NaClO_4_, used in previous work^14^) apparently removes the chemical forms of astatine with higher Rf observed to be formed over time/with increased dose on Reversed Phase TLC (Fig. 3a). This indicates that these forms of astatine are water soluble. In the process, however, an additional significant loss of activity is observed, that does not alter the shape of the Rf = 0 peak but simply decreases the area under the curve and results in a final low distribution ratio after a complex kinetic behaviour, Fig. 3b. This can be compared with previously reported solvent extraction data on the exact same system but where the Chloroform Eluate had received a significantly lower absorbed dose, displaying a different kinetic behaviour with a constant low distribution ratio^14^. This result was also confirmed and repeated in this study with a Chloroform Eluate with lower absorbed Dose, Fig. 3b. The results presented in Figs. 2 and 3 indicate that several chemical forms of astatine are combined in the Rf = 0 peak in both reversed phase and normal phase TLC at different absorbed doses and that a large fraction is water soluble. They further suggest that the complex kinetic behaviour upon solvent extraction of a Chloroform Eluate that has received a large dose can be attributed to the forms of astatine in the second reversed phase TLC chromatogram peak at Rf = 0.27.

**Figure 4:**
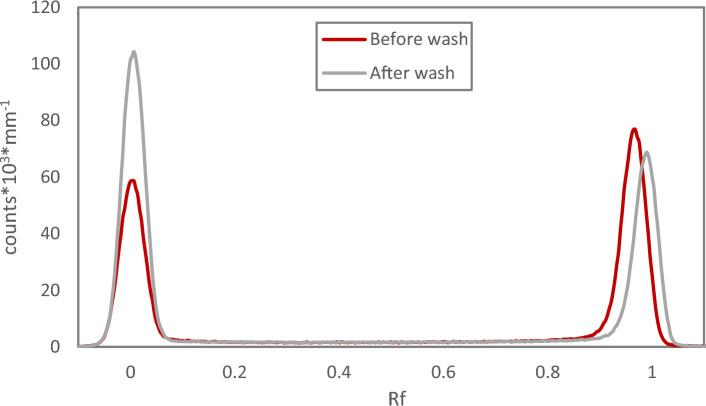
Normal phase radio-TLC analysis of dry astatine-211 from Chloroform Eluate, redissolved in fresh chloroform and subjected to multi-step washing with MilliQ water.

In a recent publication it is recommended that Chloroform Eluate after dry distillation is washed with water^15^. In the present study, simply applying equal phase volumes of MilliQ water and Chloroform Eluate and contacting briefly results in a distribution ratio of 0.02, in good agreement with the results in Fig. 3 for the NaClO_4_ solvent extraction system. This corresponds to < 3% of the activity remaining in the organic phase. Washing is common practice in some labs working with astatine if the Chloroform Eluate has been aged e.g. during transport, or when a dry vial of evaporated Chloroform Eluate is supposed to be used after being left for a longer period of time. Then a multi-step washing protocol with introduction of fresh chloroform for back-extraction of water-soluble forms of astatine is used. When applying this washing method directly on Chloroform Eluate in this study, the loss of activity from the organic phase was lower but still significant and also largely varying, Table 1. However, when first evaporating the Chloroform Eluate and redissolving the dry astatine residue in fresh chloroform directly before applying the said washing protocol, only around 10% of the activity was lost (Redissolved 1a, Table 1). This suggests that absorbed dose to the chloroform plays a significant role in the formation of water-soluble forms of astatine. The washed chloroform could again be evaporated to dryness without loss of astatine activity. The behaviour upon washing was similar if the dry vial was left for several hours before redissolution and washing (Redissolved 1b, Table 1). Normal Phase radio-TLC analysis of this redissolved astatine in fresh chloroform before and after washing show that the relation between the two (or more) forms of astatine with different mobility shifts and that the chemical form(s) of astatine at Rf = 1 seemingly account for the moderate activity losses to the water phase, Fig. 4. Upon redissolution in fresh chloroform, the chemical forms of astatine detected is seemingly similar as in Chloroform Eluate, Fig. 4 compared to Fig. 2, despite the significantly different behaviour upon water phase contact. This highlights the limitations in using TLC for detailed speciation of astatine, which is not the intention of the present work. Others have made attempts in this direction using well-defined iodine species as reference^16,17^. Attempting to use iodine as reference in the present TLC system was found not to be successful with compromised chromatograms even for as simple compounds as iodide, emphasizing the difference in chemical behaviour between astatine and iodine. If the redissolved chloroform is left for several hours before washing, then a significant loss of activity and a large variability in the results was found, despite the low dose received by the solvent compared to the original Chloroform Eluate (Redissolved 2, Table 1). This suggests that there are different mechanisms responsible for the formation of water-soluble forms of astatine in chloroform depending on the range of absorbed dose to the diluent.

**Table 1:** Multi-step washing of Chloroform Eluate and astatine-211 redissolved in chloroform with MilliQ water. Time A: Time between evaporation and redissolution. Time B: Time between redissolution and washing. Dose received by the chloroform subjected to washing. Remaining Activity % of the organic chloroform phase compared to before washing. n = 3 for redissolved 1a and n = 4 for redissolved 1b.

Type of solvent	Time A (h)	Time B (h)	Dose (kGy)	Remaining activity (%)
Eluate	–	–	3.47	65
Eluate	–	–	7.89	10
Eluate	–	–	9.63	22
Redissolved 1a	1	0	–	92.1 ± 0.7
Redissolved 1b	5–6.5	0	–	89.5 ± 3.4
Redissolved 2	1	3.5	0.062	81
Redissolved 2	1	3.5	0.062	75
Redissolved 2	1	3.5	0.140	22
Redissolved 2	21	5.5	0.092	63

### Using astatine from dry chloroform eluate

**Figure 5:**
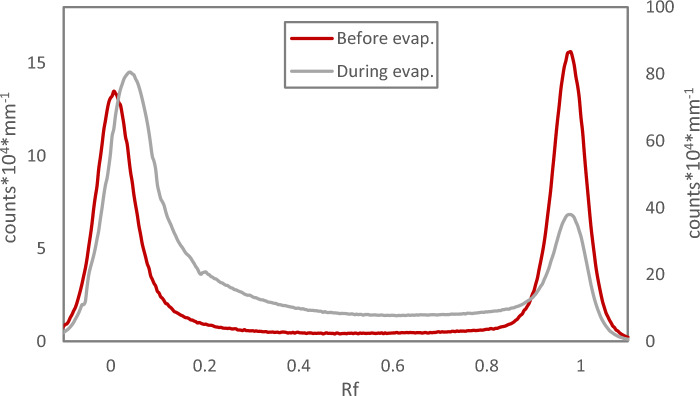
Normal phase radio-TLC analysis of astatine-211 from Chloroform Eluate before starting evaporation (activity concentration 1.2 MBq µL^−1^) and during evaporation just before reaching dryness (activity concentration *circa* 17 MBq µL^−1^). Evaporation time *circa* 3 min.

Evaporating Chloroform Eluate creating a dry residue of astatine is often used as a starting point for many different types of chemical reactions and applications, both organic and aqueous^18–20^. Trying to elucidate the actual astatine speciation in the dry form of Chloroform Eluate is naturally difficult. However, when comparing astatine in Chloroform Eluate before evaporation with the final fraction just before dryness, when *circa* 1–2 µL of solvent remain, it is obvious from Normal Phase TLC analysis that the chemical forms of astatine in the solution changes during the process, Fig. 5. The form(s) of astatine found at Rf = 0, with either higher polarity, or larger reactivity towards the Silica, becomes dominating when moving closer to the end of the evaporation process. The behaviour is seemingly similar to when Chloroform Eluate has been left for a long period of time to receive a high absorbed dose (see Fig. 2), which is consistent with the activity concentration rapidly increasing during evaporation. When redissolving dry Chloroform Eluate in fresh chloroform, as shown in Fig. 4, the relation between the different forms of astatine detected is not maintained from the near-dry form, highlighting the difficulty in analysing the actual dry species.

Potential conversion of the observed forms of astatine in near-dry Chloroform Eluate to the actual chemical reaction solution was investigated. This was done by applying various reducing and oxidizing agents commonly used within astatine chemistry to vials with Chloroform Eluate evaporated to dryness. Applying a reducing agent would aim towards forming astatide, or other At^-^ species, for use in e.g. nucleophilic astatine chemistry whereas applying an oxidizing agent would aim to form At^+^ species, predominately At(I) and/or At(III), for use in e.g. electrophilic astatine chemistry^21^.

**Figure 6:**
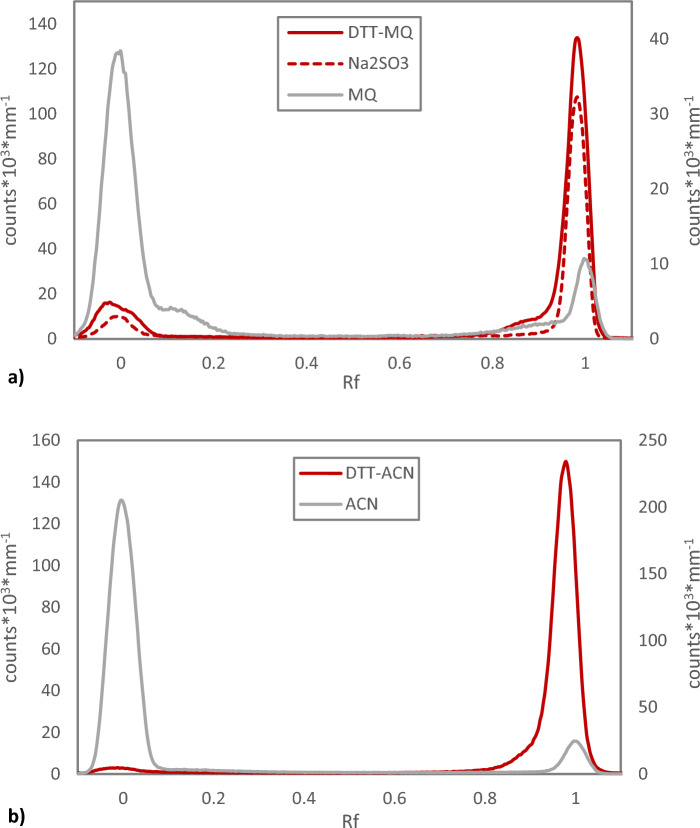
Normal phase radio-TLC comparing redissolution of dry astatine-211 from Chloroform Eluate in pure solvent to reduction using Dithiothreitol (DTT) (5 mg mL^−1^) and Na_2_SO_3_ (10 mg mL^−1^), dissolved in the same solvent. (**a**) An aqueous system with MilliQ water (MQ) as solvent. (**b**) An organic system with acetonitrile (ACN) as solvent.

When applying the commonly used reducing agents Dithiothreitol, DTT, also known as Cleland’s reagent, and sodium sulphite, Na_2_SO_3_, it is clear that astatine speciation changes to become more mobile towards Rf = 1, as compared to redissolution in the reaction solvents acetonitrile and MilliQ water alone, where activity predominately remain in the origin, Fig. 6. This indicates that reduction to an At^-^ based form of astatine is efficient.

**Figure 7:**
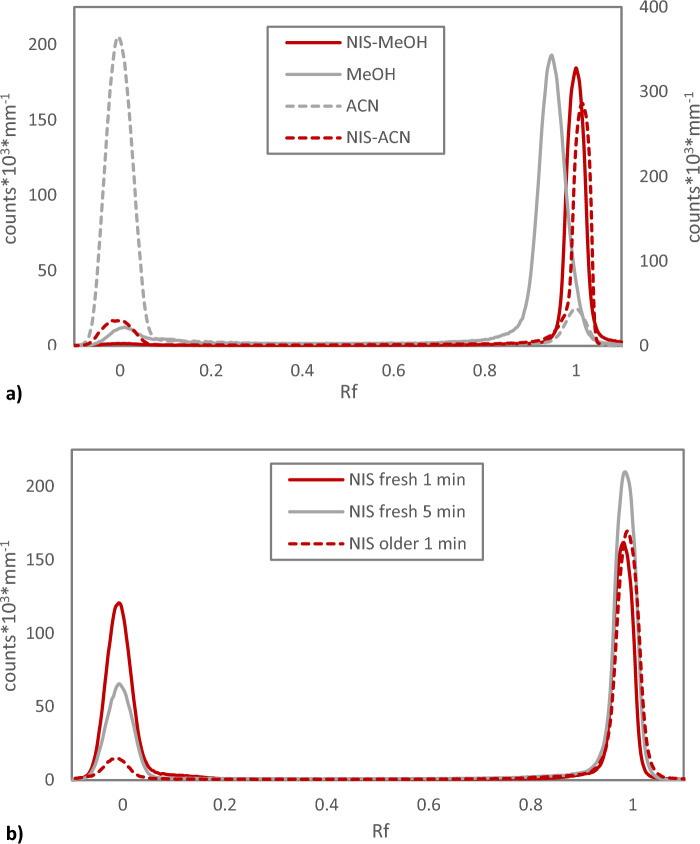
Radio-TLC analysis of astatine-211 oxidized with *N*-Iodo Succinimide. (**a**) Normal Phase TLC comparing redissolution of dry astatine-211 from Chloroform Eluate in pure methanol (MeOH) and acetonitrile (ACN) to oxidation using *N*-Iodo Succinimide (NIS) (20 µg mL^−1^ in 1% Acetic acid in methanol and acetonitrile) made > 60 min prior to the reaction with 5 min reaction time. (**b**) Normal phase radio-TLC comparing NIS oxidation (20 µg mL^−1^ in 1% acetic acid in methanol) of dry astatine-211 from Chloroform Eluate shortly after preparing the oxidation solution (< 5 min) “fresh” with different reaction times with the same solution after > 30 min “older”.

Halogenated succinimides such as *N*-chloro-succinimide (NCS) and *N*-iodo succinimide (NIS) are commonly used for oxidative astatine chemistry^22,23^. NIS proved efficient in producing an oxidized form of astatine with high mobility in the analytical system, Fig. 7a. In this case, however, also the most commonly used solvent for the application, methanol, produces a form of astatine with similar Rf. Despite this, the slight difference in Rf and shape of the peak, which is broader in the solvent, indicates that the actual chemical forms of astatine are different in the two cases. This can be confirmed when instead applying NIS in acetonitrile, as then the predominant activity peak readily shifts from Rf = 0 for the solvent to Rf = 1, Fig. 7a.

When the NIS solution in methanol was used immediately (< 5 min) after preparation, the presence of two different forms of astatine in solution was found. Both reaction time and time after preparation influence the relative amount of the form of astatine found at Rf = 0 Fig. 7b. The same behaviour can be found when analysing the solutions with the reversed phase TLC system (Supplementary Fig. S2). The NIS solution ages over time, which is evident at higher concentrations as the colour gradually changes from transparent to orange, which turns darker with time. However, preparing the NIS solution long before the reaction (> 4 h) still facilitates an efficient oxidation of the astatine.

**Figure 8:**
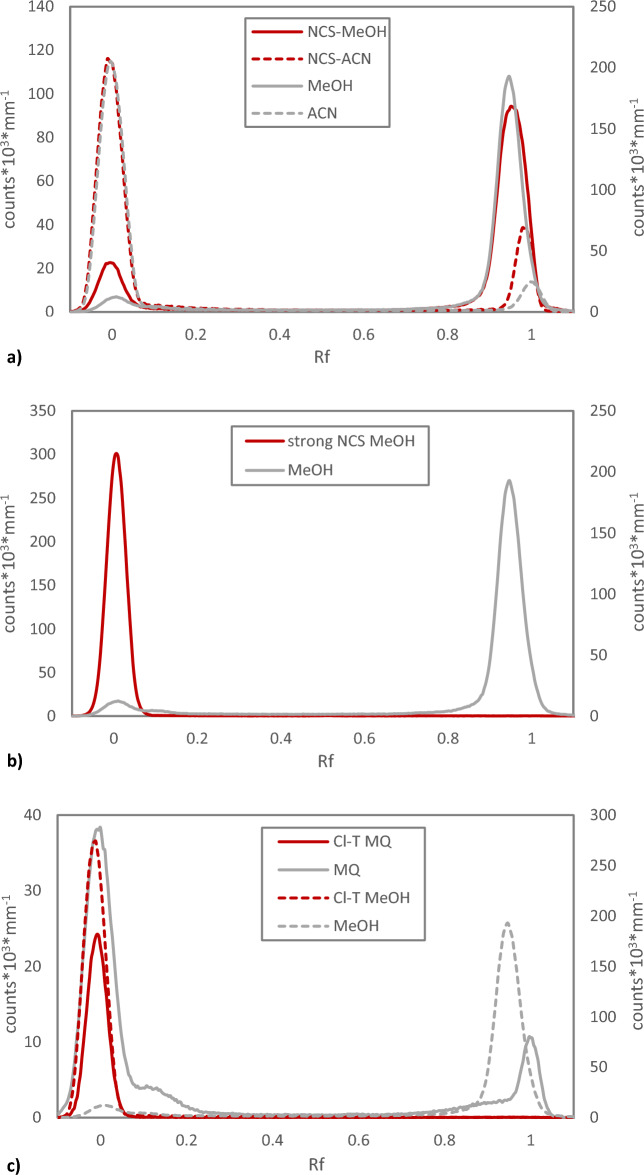
Radio-TLC analysis of astatine-211 oxidized with *N*-Chloro Succinimide and Chloramine T. (**a**) Normal Phase radio-TLC comparing redissolution of dry astatine-211 from Chloroform Eluate in pure methanol (MeOH) and acetonitrile (ACN) to oxidation using *N*-Chloro Succinimide NCS (100 µg mL^−1^ in 1% acetic acid in methanol and acetonitrile). (**b**) Normal phase radio-TLC comparing redissolution of dry astatine-211 from Chloroform Eluate in pure methanol to high concentration NCS oxidation (5 mg mL^−1^ in 1% Acetic acid in methanol). (**c**) Normal Phase radio-TLC comparing redissolution of dry astatine-211 from Chloroform Eluate in MilliQ water (MQ) and methanol (MeOH) with oxidation using Chloramine-T (Cl-T, 5 mg mL^−1^) in the same solvents.

When applying NCS to dry astatine from Chloroform Eluate, the behavior was found to be very different compared to NIS. Using NCS in a five times higher concentration compared to NIS did not induce any apparent oxidation or changes in the chromatogram, compared to applying the solvents alone, Fig. 8a. When increasing the NCS concentration even further, a shift in detected forms of astatine cold be facilitated, but contrary to NIS, and the application of reducing agents, this form of astatine was found at Rf = 0, Fig. 8b. A significantly increased polarity of the resulting compound compared to oxidation with NIS or reduction is less likely than the formation of an interhalogen specie that tend to react with the TLC stationary phase.

Another chlorine-based oxidation agent, Chloramine-T, has historically been used for oxidizing astatine recovered from methanol^24^. Application of chloramine-T was in this study, just like NCS, found to result in a chemical form of astatine detected at Rf = 0. Despite not shifting the main Rf as compared to the solvent, when applied in MilliQ water, the peak shape and overall appearance of the chromatogram suggests that the chemical forms are different in the two cases. This is consistent to when applying the oxidizing agent in methanol, where the peak then shifts from Rf = 1 in the solvent to Rf = 0, Fig. 8c.

## Discussion

When evaporating Chloroform Eluate to dryness it is important to stop the gas flow directly when the liquid phase has been removed not to lose activity. Applying a small amount of heat and a slightly increased flow of nitrogen can be used to speed up the evaporation process without loss of activity. Stopping promptly after the point of dryness is even more important when high astatine activities are eluted in small volumes of chloroform. This as the high activity concentration and absorbed dose to the solvent results in a larger loss of activity in a shorter amount of time upon prolonged N_2_ flow over the dry astatine residue. Considering efforts to increase astatine production yields from the cyclotrons and production for clinical applications this is very important in order to minimize activity losses, both from a radiation protection point of view as well as from a patient perspective, to ensure enough activity remain to create sufficiently large patient doses.

Working with a dry vial of astatine can be considered safe as any loss of activity without a directed gas flow into the vial is found to be minor, even when a lid is not applied. This would of course depend on the geometry and size of the vial as losses would become slightly higher in larger vials with large openings as the natural turnover of the atmosphere in the vial would increase.

It is advisable to use fresh and properly stored chloroform to form the Chloroform Eluate in order to reduce loss of activity and maintain radiation safety. Photochemical degradation of chloroform form phosgene, hydrogen chloride as well as chlorine^25^. It is likely that one or several of these species are relevant for the volatilization of astatine upon evaporation of Chloroform Eluate formed when using aged chloroform. As absorbed dose to the solvent reduces this effect it can be assumed that upon irradiation the photochemical degradation products responsible for volatilizing astatine are consumed by reactions with radiolysis products of chloroform. The importance of chlorine species in the stabilization of astatine is evident when replacing chlorine with bromine in the form of di-bromo methane. A significant loss of activity is observed upon evaporation of this solvent, and it is therefore not advised to use brominated diluents for astatine recovery.

Analysing old Chloroform Eluate that had received a high absorbed dose with different radio-TLC systems showed that speciation changed compared to fresh Chloroform Eluate. Whether this change in speciation influences the reactivity in downstream chemistry or not is currently under investigation and will be presented in future work.

In a recent publication, washing of Chloroform Eluate with water is recommended in order to remove HCl formed by radiolysis of the chloroform^15^. From the results in the present study it can, however, be concluded that this is not always advisable as this may cause major losses of activity from the organic phase. Also, the way in which the washing is performed plays a significant role in how much activity that is lost from the Chloroform Eluate. One explanation besides absorbed dose, to the large discrepancy in results between different laboratories could be differences in the dry distillation and subsequent astatine solvation process, where the amount of oxygen in the carrier gas may vary, influencing the formation of radiolysis products in the chloroform. Both solvent extraction and washing results in this study show that astatine speciation in chloroform do change upon contact with an aqueous phase. These processes are, however, both complex and unreliable as several different mechanisms seem to be involved in the formation of water-soluble forms of astatine at different levels of absorbed dose to the diluent. In all cases, the chloroform that has been subjected to aqueous phase contact seemingly can be evaporated to dryness without loss of the present astatine activity. This is most likely possible due to the very low solubility of water in chloroform, 0.093%^26^. However, if a droplet of water is visible in the Chloroform Eluate before evaporation, then activity can be observed to be lost when the chloroform is evaporated and only the small water fraction remains. This can be confirmed when evaporating astatine dissolved in other organic solvents, such as acetonitrile or ethyl acetate, with a significant water content, as when the water fraction in the solvent increases towards the end of the evaporation, a significant, up to 30%, loss of astatine activity can be observed.

Using astatine from a dry residue after evaporating Chloroform Eluate is an efficient starting point for different types of astatine chemistry. In this study TLC was used for comparative analysis to detect changes in the chemical forms of astatine when applying different oxidizing and reducing agents to dry Chloroform Eluate. To detect such changes and differentiate between different forms of astatine, however, place demands on both the analytical system and reaction conditions chosen, especially if the user does not have access to a TLC scanner but instead evaluate TLC’s by cutting the plates and measuring the activity. From the results in this study, it is evident that the choice of oxidizing/reducing agent as well as the reaction conditions play a significant role in the resulting chemical forms of the astatine. Utilizing chlorine based oxidizing agents rendered significantly different results as compared to using an Iodine based oxidizing agent. Both oxidation using NIS and reduction using DTT and Na_2_SO_3_ resulted in chemical forms of astatine that were readily mobile in the applied TLC system, whereas application of NCS or Chloramine T resulted in forms of astatine that did not move from the origin. Astatine in Chloroform Eluate is, as shown in previous work, most likely a mixture of different species involving chlorine containing radiolysis products of chloroform^14^. This may be the reason why oxidation of astatine using chlorine-based agents renders this somewhat unexpected result. How the speciation of astatine in these different reducing or oxidizing starting solutions influence the astatination yield in downstream chemistry must, however, be assessed for every single case.

## Methods

All aqueous solutions were prepared using ultrapure water, MilliQ (> 18.2 MΩ*cm). Nitrogen gas used for evaporation and dry distillation was Scientific Nitrogen 6.0 HiQ^®^, 99.9999 mol% N2. Chloroform was of Analytical grade, EMSURE^®^, stabilized with 0.6–1% Ethanol. All other chemical reagents used were of analytical grade or higher. All presented data have been decay corrected in order to separate natural decay of astatine-211 and activity losses due to chemical processes.

### Astatine production and recovery

Astatine-211 was produced at Copenhagen University Hospital by 28–29 MeV alpha particle bombardment (MC32 Scanditronix cyclotron) of an aluminium target (0.5 × 27 × 30 mm) coated with bismuth (24 ± 2 mg*cm^−2^) and aluminum (0.4–0.5 mg*cm^−2^). 4 h irradiation yield 1–1.4 GBq of astatine-211 EOB. The irradiated target is transported by road (3.5–4 h) to Gothenburg, Sweden and upon arrival the astatine-211 (*circa* 800 MBq) is recovered using automatic dry distillation (Atley C100). The activity is eluted using chloroform (*circa* 0.5–1 mL) rendering an activity concentration of 0.7–2 MBq*μL^−1^ of chloroform. The resulting astatine-211 chloroform solution, named Chloroform Eluate, was then used as-is or, evaporated to dryness or subjected to chemical reactions in dry or wet form.

### Activity analysis

Astatine activities were measured using two different types of available dose calibrators, VDC-505/VIK-202 from Comecer or CRC15 from Capintec with conversion factors for astatine. During one single experiment the same dose calibrator was always used for comparative measurements.

### Absorbed dose calculations

To calculate the absorbed dose, D, in Gray to a solvent containing astatine the following equation is used:

1 $${\text{D}} = \left( {\left( {\left( {{{\text{A}}_0}/\lambda } \right)*\left( {1 - {{\text{e}}^{( - \lambda *{\text{t}})}}} \right)} \right)*{\text{E}}} \right)/\left( {{\text{V}}*\rho } \right)$$

where A_0_ is the astatine activity at t = 0, t is the time from t = 0 until calculation of the absorbed dose, E is the energy from one decay of astatine in Joule, λ is the decay constant for astatine, V is the volume of the solvent and ρ is the density of the solvent.

Where applicable the time it takes to evaporate the solvent has been excluded from the dose calculations, as this in all cases is < 5 min.

### Evaporation

Astatine-211 recovered in chloroform after dry-distillation, Chloroform Eluate (5–100 μL), was evaporated in a fume hood until dryness, unless otherwise stated, by placing a portion of the chloroform in a Screw Top Tapered (1.1 mL) or Flat Bottom (2 mL) vial without a lid under a stream of nitrogen (130–220 mL h^−1^) from a 200 µL pipette tip. The pipette tip was placed above the surface of the chloroform and connected to a gas flow meter and the available house-gas outlet via silicon tubing.

This process was, unless otherwise stated, performed at room temperature and took between 0.3–5 min depending on the volume of Chloroform Eluate and the vial applied. The same type of vial was always used in the same set of experiments. When Chloroform Eluate was formed with aged chloroform before evaporation studies, this chloroform had been sitting in a 20 mL clear glass container for > 3 weeks at room temperature exposed to light from fluorescent tubes during working hours.

Dry portions of astatine-211 from Chloroform Eluate were also redissolved in di-bromo methane and after different periods of time small portions (3 × 20 μL) from these solutions were removed and once again subjected to evaporation as described above. It should be noted that when working with dibromo methane and measuring astatine activity by the 75–93 keV X-ray emission of Po-211, a significant amount of attenuation was observed in the solvent, resulting in the need to measure activity before adding the brominated solvent and after evaporation not to get false positive results.

### Solvent extraction

Chloroform Eluate was subjected to solvent extraction by contacting equal phase volumes (80 µL) of the organic phase with 0.1 M NaClO_4_ on an IKA VIBRAX mechanical vortex shaker in glass vials with screw top lid (2 mL). For kinetic experiments, equal volume samples (5 µL) of the two phases were removed after different periods of time for activity distribution analysis. Results are presented in terms of astatine distribution ratios, determined by measuring the activity in both phases, dividing the activity in the organic phase with the activity in the aqueous phase.

### Washing

A portion, Y, between 50–500 µL, of Chloroform Eluate or redissolved Chloroform was contacted with 0.4*Y µL of MQ water (18 MΩ*cm) for 1 min. The organic phase was then carefully removed. The remaining aqueous phase was then briefly contacted with 0.5*Y µL of fresh chloroform, which subsequently was carefully removed and combined with the first organic phase fraction. Activity of the organic phase was measured before and after washing as well as the activity of the aqueous phase.

### Oxidation/reduction

To a 1.1 mL tapered screw top vial with dried down Chloroform Eluate, 50 µL of reducing or oxidizing agent was added. After a contact time of 5 min, unless otherwise stated, samples were removed for TLC analyses (see separate section). Di-thiothreitol, DTT, was applied in a concentration of 5 mg mL^−1^ in both acetonitrile and MilliQ water. Sodium sulfite, Na_2_SO_3_, was applied in MilliQ water in a concentration of 10 mg mL^−1^. *N*-Iodo succinimide, NIS, was applied in a concentration of 20 µg mL^−1^ in methanol with 1% acetic acid and acetonitrile. *N*-chloro succinimide, NCS, was applied in a concentration of 100 µg mL^−1^ in methanol with 1% acetic acid and acetonitrile as well as in 5 mg mL^−1^ in methanol with 1% acetic acid. Chloramine T was applied in a concentration of 5 mg mL^−1^ in MilliQ water and methanol.

### Thin layer chromatography (TLC)

TLC plates with aluminum backing (Normal Phase: Silica Gel 60 F254 or Reversed Phase: Silica gel 60 RP-18 F254S, Supelco) were spotted with 1-(2×)3 µL of the relevant analyte resulting in analytical amounts of activity, typically < 1 MBq. After drying, the plates were placed in conical screw cap poly propylene vial (50 mL) with 1–1.5 mL mobile phase (Normal phase: Acetonitrile, Reversed Phase: Acetonitrile/water (80:20)). When the front had travelled *circa* 7 cm the plates were dried and analyzed for 5–10 min with a digital TLC scanner with alpha detection (miniGita dual, Elysia Raytest, GinaX software) at a 2 or 4 mm detector distance. Spotting volume and scanning settings were adjusted to fit the analyte activity. The mobile phase was measured after analysis to ensure minimal bleeding of activity from the TLC plate.

### Supplementary Information

Supplementary Figures.

## Data Availability

All relevant data generated or analyzed during this study are included in this published article.

## References

[1] Sgouros G (2010). MIRD Pamphlet No. 22 (abridged): Radiobiology and dosimetry of alpha-particle emitters for targeted radionuclide therapy. J. Nucl. Med..

[2] Palm S (2016). A biokinetic modeling and dosimetry for optimizing intraperitoneal radioimmunotherapy of ovarian cancer microtumors. J. Nucl. Med..

[3] Sofou S (2008). Radionuclide carriers for targeting of cancer. Int. J. Nanomedicine.

[4] Feng Y, Zalutsky MR (2021). Production, purification and availability of (211)At: Near term steps towards global access. Nucl. Med. Biol..

[5] Takahashi N (2002). Boiling points of the superheavy elements 117 and 118. J. Radioanal. Nucl. Chem..

[6] Haynes WM, Haynes WM (2014). Melting, boiling, triple and critical points of the elements. CRC Handbook of Chemistry and Physics.

[7] Guerard F (2021). Advances in the chemistry of astatine and implications for the development of radiopharmaceuticals. Acc. Chem. Res..

[8] Pozzi OR, Zalutsky MR (2005). Radiopharmaceutical chemistry of targeted radiotherapeutic’s, part 1: Effects of solvent on the degradation of radiohalogenation precursors by At-211 alpha-particles. J. Nucl. Med..

[9] Zalutsky MR, Pruszynski M (2011). Astatine-211: Production and availability. Curr. Radiopharm..

[10] Andersson H (2009). Intraperitoneal alpha-particle radioimmunotherapy of ovarian cancer patients: Pharmacokinetics and dosimetry of (211)At-MX35 F(ab')2–a phase I study. J. Nucl. Med..

[11] Hallqvist A (2019). Intraperitoneal alpha-emitting radioimmunotherapy with (211)At in relapsed ovarian cancer: long-term follow-up with individual absorbed dose estimations. J. Nucl. Med..

[12] Lindegren S, Back T, Jensen HJ (2001). Dry-distillation of astatine-211 from irradiated bismuth targets: A time-saving procedure with high recovery yields. Appl. Radiat. Isot..

[13] Albertsson P (2022). Astatine-211 based radionuclide therapy: Current clinical trial landscape. Front. Med. (Lausanne).

[14] Aneheim E (2019). Towards elucidating the radiochemistry of astatine—Behavior in chloroform. Sci. Rep..

[15] Ghalei M (2022). How radiolysis impacts astatine speciation?. Radiat. Phys. Chem..

[16] Nishinaka I, Hashimoto K, Suzuki H (2018). Thin layer chromatography for astatine and iodine in solutions prepared by dry distillation. J. Radioanal. Nucl. Chem..

[17] Nishinaka I, Hashimoto K, Suzuki H (2019). Speciation of astatine reacted with oxidizing and reducing reagents by thin layer chromatography: Formation of volatile astatine. J. Radioanal. Nucl. Chem..

[18] Timperanza C, Jensen H, Back T, Lindegren S, Aneheim E (2023). Pretargeted alpha therapy of disseminated cancer combining click chemistry and astatine-211. Pharmaceuticals-Basel.

[19] Berdal M (2021). Investigation on the reactivity of nucleophilic radiohalogens with arylboronic acids in water: Access to an efficient single-step method for the radioiodination and astatination of antibodies. Chem. Sci..

[20] Maingueneau C (2022). At-211 and I-125-labeling of (hetero)aryliodonium ylides: Astatine wins again. Chem. Eur. J..

[21] Guerard F, Gestin JF, Brechbiel MW (2013). Production of [(211)At]-astatinated radiopharmaceuticals and applications in targeted alpha-particle therapy. Cancer Biother. Radiopharm..

[22] Vaidyanathan G (2021). Synthesis and preliminary evaluation of (211)At-labeled inhibitors of prostate-specific membrane antigen for targeted alpha particle therapy of prostate cancer. Nucl. Med. Biol..

[23] Palm S (2021). Evaluation of therapeutic efficacy of (211)At-labeled farletuzumab in an intraperitoneal mouse model of disseminated ovarian cancer. Transl. Oncol..

[24] Orlova A (2000). Closo-dodecaborate (2-) anion as a potential prosthetic group for attachment of astatine to proteins. Aspects of the labelling chemistry with Chloramine-T. J. Labelled Compd. Radiopharm..

[25] Teipel J (2022). An easy and reliable method for the mitigation of deuterated chloroform decomposition to stabilise susceptible NMR samples. Chemistry (Basel).

[26] Marcus I, Rydberg J, Cox M, Musikas C, Choppin GR (2004). Solute solvent interactons. Solvent Extraction Principle and Practice.

