# Supplementary material for: Behaviour, use and safety aspects of astatine-211 solvated in chloroform after dry distillation recovery
Source: Sci Rep. 2024 Apr 27;14:9698. doi: 10.1038/s41598-024-60615-4 (PMC11055885; doi:10.1038/s41598-024-60615-4)
Supplement: Supplementary file 1 — Supplementary Figures. [file 41598_2024_60615_MOESM1_ESM.docx]

Supplementary material to:

Behaviour, Use and Safety Aspects of Astatine-211 Solvated in Chloroform after Dry Distillation Recovery

Emma Aneheim*^1,2^, Ellinor Hansson^1,3^, Chiara Timperanza^1^, Holger Jensen^4^, Sture Lindegren^1^

^1^Department of Medical Radiation Sciences, Institute of Clinical Sciences, Sahlgrenska Academy, University of Gothenburg, SE41345, Gothenburg, Sweden. ^2^Region Västra Götaland, Sahlgrenska University Hospital, Department of Oncology, SE41345, Gothenburg, Sweden. ^3^Atley Solutions AB, SE41133, Gothenburg, Sweden. ^4^PET and Cyclotron Unit, Copenhagen University Hospital, KF3982, Copenhagen, Denmark.

*email: emma.aneheim@radfys.gu.se

Figure S1. Retained astatine-211 activity as a function of absorbed dose to solvent upon redissolution of dry astatine-211 from Chloroform Eluate in dibromo methane and subsequent evaporation of the brominated solvent after different contact times and hence absorbed doses.

Figure S2. Reversed phase radio-TLC (mobile phase 80:20 acetonitrile/water) on N-Iodo Succinimide (NIS) oxidation (20 µg/mL in 1% Acetic acid in methanol) of dry astatine-211 from Chloroform Eluate. Comparing NIS solutions made immediately before oxidation, “fresh” and after > 15 minutes.
